# What Can We Learn from -Omics Approaches to Understand Clubroot Disease?

**DOI:** 10.3390/ijms23116293

**Published:** 2022-06-04

**Authors:** Jutta Ludwig-Müller

**Affiliations:** Faculty of Biology, Technische Universität Dresden, 01062 Dresden, Germany; jutta.ludwig-mueller@tu-dresden.de

**Keywords:** *brassica* host, clubroot, effector, genome, metabolome, microbiome, *Plasmodiophora brassicae*, proteome, transcriptome

## Abstract

Clubroot is one of the most economically significant diseases worldwide. As a result, many investigations focus on both curing the disease and in-depth molecular studies. Although the first transcriptome dataset for the clubroot disease describing the clubroot disease was published in 2006, many different pathogen–host plant combinations have only recently been investigated and published. Articles presenting -omics data and the clubroot pathogen *Plasmodiophora brassicae* as well as different host plants were analyzed to summarize the findings in the richness of these datasets. Although genome data for the protist have only recently become available, many effector candidates have been identified, but their functional characterization is incomplete. A better understanding of the life cycle is clearly required to comprehend its function. While only a few proteome studies and metabolome analyses were performed, the majority of studies used microarrays and RNAseq approaches to study transcriptomes. Metabolites, comprising chemical groups like hormones were generally studied in a more targeted manner. Furthermore, functional approaches based on such datasets have been carried out employing mutants, transgenic lines, or ecotypes/cultivars of either *Arabidopsis thaliana* or other economically important host plants of the *Brassica* family. This has led to new discoveries of potential genes involved in disease development or in (partial) resistance or tolerance to *P. brassicae*. The overall contribution of individual experimental setups to a larger picture will be discussed in this review.

## 1. Introduction

**Figure 1:**
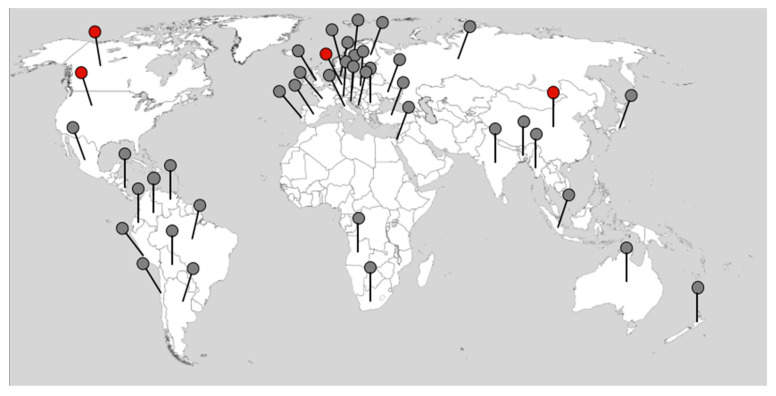
The worldwide distribution of *P. brassicae* (based on references until the beginning of 2022). The dots show all countries where the occurrence of *P. brassicae* was reported. The red dots indicate the geographical origin of those where sequence information is available (based on information beginning in 2022). The map is from https://d-maps.com/carte.php?num_car=13180&lang=de (accessed on 25 February 2022).

**Figure 2:**
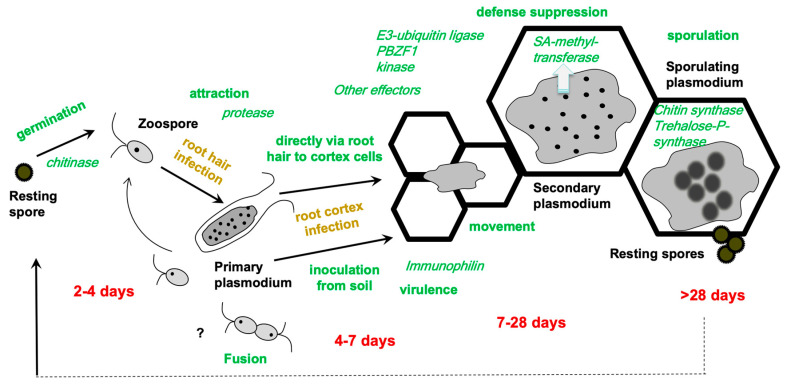
The most important life stages are given together with the approximate time points of their occurrence based on different host plants and/or isolates or experimental conditions. Typical time intervals are indicated below in red, in green the various processes that are related to *P. brassicae* colonization are shown, and in brown, the two different phases of the life cycle are indicated. In italics, some important *P. brassicae* genes/proteins are given for which a function has been experimentally shown. The identification and characterization of the effectors are described in Section 4.1.

Understanding the interaction of host plants with pathogens is crucial for determining their life cycle but also for developing control strategies [1]. Furthermore, the host response in terms of metabolism and/or development is critical. While traditional phytopathology approaches and microscopy can be used to monitor the interaction, molecular analyses are needed for a more in-depth view. Specific transcripts, proteins, and metabolites are important for a certain stage of disease development or s combination of host and pathogen properties. When mutants are available, e.g., for the model plant *Arabidopsis thaliana*, functional investigations can also be performed [2,3]. Additionally, the use of susceptible and resistant *Brassica* cultivars for transcriptome and proteome analyses has been included (e.g., [4,5,6]). Clubroot disease is one of the most ubiquitous diseases today, having recorded breakouts on all continents [1,7], and it can be found in a plethora of countries worldwide (Figure 1). The red dots indicate the geographical origin of spore isolates/pathotypes that have been sequenced up to date. Many studies employing large datasets to understand the biology of this soilborne phytopathogenic protist have been reported. However, when looking into large sets of data, it can be difficult to extract the key message at a glance, which is what this review aims to do, and in some cases, this might lead to an oversimplified view. The time point of analysis is critical for the expected results since the life cycle of this biotrophic pathogen is so complex. Therefore, the specific and important stages will be described in Section 2 and related to information from potential effectors of *P. brassicae* that have been identified so far (Figure 2). The aim of this review is to evaluate how much knowledge can be obtained from published genomes and other -omics datasets. Additionally, the data should be made available in a way that allows for comparisons across different datasets utilizing, for example, the same host plant.

## 2. How to Integrate -Omics Data into the Life Cycle of *Plasmodiophora brassicae*

Although the life cycle has been detailed in many publications over the past two centuries (e.g., [8,9]), some details are still enigmatic (Figure 2). Recently, the combination of staining methods and microscopy has led to an improved life cycle with the addition of some more, previously questioned, phases [10]. The development of *P. brassicae* in its host is important to understand since it cannot be cultivated outside its host. In addition, only a few stages, i.e., resting spores and the biflagellated zoospores, exist outside of the host plant in the soil. In susceptible interactions, the stages are the same for all combinations of pathogen isolates and host plant, while they can differ in resistant interactions [11,12]. Aside from the stages, the timing of a particular developmental step within host plants can also differ depending on the virulence of the pathotype of *P. brassicae* in question. As a result, the time periods in Figure 2 are only estimates for the various associated stages. The life cycle starts with the germination of the resting spore, which transforms into a mobile zoospore. The latter attaches to a root hair [9] or—as recent evidence has also suggested—directly to the epidermis of a root [10]. Within the host cells, the zoospores encyst and grow into a multinucleate plasmodium. During the primary infection, the root hairs are colonized, while during the secondary infection the colonization of the complete cortex occurs. Finally, the multinucleate plasmodia mature into resting spores that are released into the soil when the tissue decomposes.

As previously stated, some stages remain unknown, such as karyogamy and meiosis, colonization of the cortex, and the movement of *P. brassicae* in the tissue and/or into the vasculature. The latter raises the question of how *P. brassicae* is restricted to the root and hypocotyl of the host plant. Although this is not the focus of this review, a few points will be added since such questions might be addressed using a combination of -omics and microscopy. Liu et al. [10] described a pair of zoospores that appear to have fused. However, the production of single spore isolates (SSI) [13,14] implies that two mating types are not required, but they do not rule out the possibility of a fusion of the same mating type. Microscopically, dikaryotic myxamoeba-like structures have also been observed in young colonized root tissue, supporting the latter idea [9]. The invasion of the root cortex is the next enigmatic step. Either the zoospores enter via the passage of a root hair to a cortex cell, or via an unknown route from the soil. Some microscopic evidence has been provided for the former [11], but as zoospore multiplication during primary infection is also a hallmark, the second route must be considered as well. While a mechanical mechanism after zoospore attachment for its protoplast injection into the root hair via a so-called Rohr and Stachel apparatus has been established [15], anything analogous to cortex invasion is still missing. The small plasmodia seem to move through the cortex after penetration [16,17,18,19] since in the beginning they are only located at the periphery, but they are rapidly found in inner cells adjacent and even within the vasculature [18]. The division of the plasmodia is accompanied by the division of host cells, resulting in clusters of infected cells, and finally colonization of the entire cortex [20,21]. The plasmodia-harboring cells elongate to provide additional space for the plasmodia to develop into resting spores [21].

## 3. What Has Been Compared?

The first report on a gene from *P. brassicae* was published in 1999, while molecular biology using individual genes and/or mutants had begun earlier. However, the gene sequence of this gene was incomplete and had no annotated function [22]. More genes were later discovered utilizing differential expression techniques such as cDNA libraries [23,24,25,26]. In the early years before the reference genome of *P. brassicae* had been published [27], there were reports on -omics approaches for the host–pathogen interaction, in the beginning only concentrating on the plant side [2,3]. Such datasets are of limited value if no functional analyses, such as using different ecotypes, cultivars, or mutants can be included. Since microarrays only produce plant sequences, information on *P. brassicae* sequences was not required for these datasets, but it would have been useful for subtractive libraries. Bulman et al. [23,28] and later Sundelin et al. [26] used a suppression subtractive hybridization strategy to identify a larger number of new *P. brassicae* genes. Dot-blot analyses of over 100 individual *P. brassicae* sequences were combined with qPCR to investigate their expression levels during the development of primary and secondary zoospores of *P. brassicae* [29] (Table 1).

**Figure 3:**
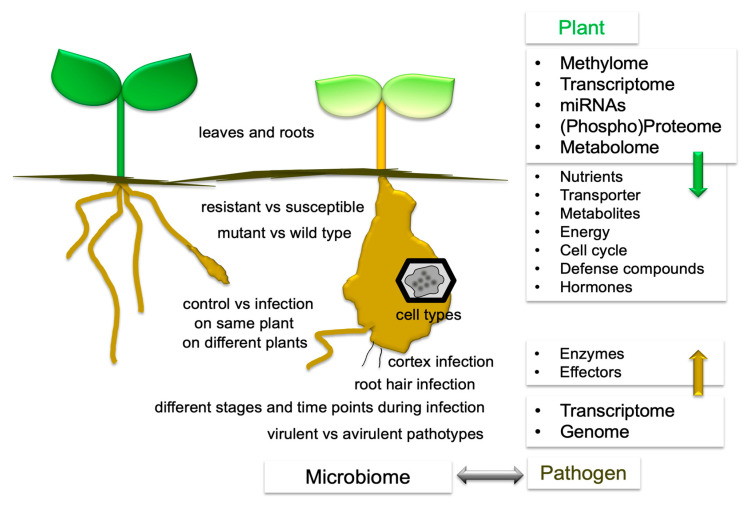
The experimental setups used for -omics studies with *P. brassicae*, as well as a summary of the results of the respective datasets. For either plant or pathogen, the -omics techniques are compiled on the right side. The results are partially integrated into a plant model together with the different genetic levels of regulation (see Figure 4).

A compilation of the comparisons from several -omics datasets has been created (Figure 3). The term transcriptome covers all methods for the analysis of differential gene expressions such as ESTs, (subtractive) cDNA libraries, microarrays, and RNAseq. Furthermore, miRNAs analysis was carried out [36]. Single cell analyses were performed using laser microdissection coupled to microarray analysis [32]. Only a few experiments reported transcriptomes [37,38] or (targeted) metabolomes [39] of root and leaf tissues. Comparisons have been made for: 1. control vs. infection on different plants or on the same plant, 2. different time points, 3. leaves and roots, 4. resistant vs. susceptible plants, 5. mutant plants vs. wild type, and 6. different cell types [2,5,20,32,35]. For more references on -omics approaches see Section 5, 6 and 7. On the plant side, there are nutrients, transporters, metabolites, energy, cell cycle, defense compounds, and hormones, as well as enzymes and effectors for the pathogen. Epigenetic modifications were also investigated [40]. The soil microbiota also contribute to the outcome of the clubroot disease and were therefore studied in more detail. Analyses were done in either one condition [41], by comparing symptomatic vs. asymptomatic roots in the same field [42], resistant and susceptible cultivars [43], rotation patterns [44], or treatments with fungicides or biocontrol agents [45,46,47], and also virulent vs. avirulent pathotypes of *P. brassicae* on one host [48]. Only a few “multi-omics” approaches have been carried out, among them hormones and proteomes [3]. For the analysis of lipids, genome, transcriptome, proteome, and metabolome data were combined [49]. The genomic information for *P. brassicae* has also been translated into knowledge for the life cycle [50]. In addition to exploring individual datasets, analyzing already deposited data for a different research subject, such as done by [51], would be possible.

## 4. *Plasmodiophora brassicae* Genomes across the Globe

Since there was insufficient plasmodiophorid genomic information prior to 2015, scientists naturally focused on the plant side of the interaction [52]. The sequencing effort granted to the rhizaria supergroup remains insignificant by comparison to other eukaryotic groups [53,54]. In addition to plasmodiophorids, only three genomes from other rhizaria protists are available: the Chlorarachnea *Bigelowiella natans* [55], the Foraminifera *Reticulomyxa filosa* [56], and the Imbricatea *Paulinella micropora* [57]. An update on the present status of other plant pathogenic plasmodiophorids is given in Section 9.

Before the first reference genome sequence was available, analyses of genome structures were performed via PCR amplification of larger genomic DNA fragments and sequencing. For example, such investigations led to a conclusion concerning the number and size of introns in *P. brassicae* [28]. Pulse field gel electrophoresis combined with hybridization to marker sequences were employed to characterize spore isolates separated by the host, followed by single spore isolates. They also revealed an approximate number of chromosomes for the ca 25 MB size of the *P. brassicae* genome [13,58,59,60].

The first genome sequence of the German SSI e3 was released as the “reference genome” in 2015 [27], then re-sequenced in 2019 [61], followed by Canadian [62] and Chinese [63] single spore isolate (SSI) sequences, so that different parts of the world are up to now already covered (Figure 1). The e3 genome was re-sequenced using PacBio sequencing, to provide more structural information [61]. Additionally, another sequence from China was added [64]. The number of genomes in the NCBI database has increased to 50 since then (accessed 2 February 2022) and is in the majority from field isolates [65,66]. Furthermore, the mitochondrial sequence has been described in detail by now [61,67]. Differences in the size and numbers of the mitochondrial genomes seem to be influenced by the sequence technologies utilized as well as the use of two different isolates [61,67]. The possibility of using genome sequences to identify putative effectors has been reviewed by Schwelm and Ludwig-Müller [66] and Perez-Lopez et al. [68].

### 4.1. Effector Candidates

Bioinformatic approaches based on accessible genomes were used to identify putative effector candidates [27,68] that predict either signal peptides or properties known from other organisms. Several hundred effector genes were predicted by this approach in the genomes currently available, mostly from the European SSI e3 or the Canadian SSI Williams pathotype P3 [27,62,68]. The model (Figure 2) incorporates what is known about *P. brassicae* proteins at different stages of the life cycle, and Table 2 summarizes the individual characterized *P. brassicae* proteins.

Effectors are important mediators for the pathogens to regulate the defense response of the respective host [69], but what criteria must a protein meet to be classified as an effector? For example, a protease, Pro1, from *P. brassicae* was found to be involved in colonization [70]. It came from a screen of differentially expressed genes done by Bulman et al. [23,24], and this dataset was also the source for the SABATH-type methyltransferase PbBSMT [71]. If knocking out this protease results in loss of colonization, it is unquestionable a pathogenicity factor, but strictu sensu it is not an effector that would be involved in defense suppression. Another way to learn more about the roles of *P. brassicae* proteins is to express them heterologously in other plant pathogenic fungi. A putative cyclophilin gene (*PbCyp3*), a member of a larger family of 20 genes in the e3 genome, was expressed in *Magnaporthe oryzae* [72] for such a functional analysis. The strain of *M. oryzae* lacked the homologous gene, and the PbCyp3 protein could complement the mutant. This resulted in higher virulence on rice plants compared to the mutant strain lacking immunophilin [72]. The protein may not be characterized as an effector that suppresses plant defense because it restored the virulence in its heterologous host.

The first effector for which an in vitro function was shown, converts the defense compound salicylic acid to its methylester when expressed in *E. coli* [71], and Me-SA is better transported in infected *A. thaliana* inflorescences. The protein was coined PbBSMT and has homology to plant SABATH-type methyltransferases [71]. The gene sequence came from the dataset of differentially expressed genes that also revealed *Pro1* [23,24]. It was highly expressed in the first *P. brassicae* genome/large-scale transcriptome of the single spore isolate e3 [27]. The high expression of *PbBSMT* was found throughout the life cycle in all stages [17,71]. *PbBSMT* mRNA was strongly associated with plasmodia as shown by RNA-FISH [17]. In addition, *PbSBMT* was among the highest expressed genes in a single root transcriptome assay [33]. The predicted signal peptide was later demonstrated to work in a heterologous yeast system [73]. The subcellular localization of *PbBSMT* in tobacco revealed its presence within the host cell [73]. Furthermore, transgenic *A. thaliana* plants overexpressing *PbBSMT* exhibited a higher Me-SA vs. SA content and were more susceptible to other pathogens [73,74]. The *PbBSMT* transcript was downregulated in roots of transgenic plants showing tolerance to clubroot infection by overexpression of an *A. thaliana* mitochondrial protein that provides constitutive SA response [51,75].

The *P. brassicae* e3 genome contains 139 putative E3 ubiquitin ligases, of which 115 show the conserved RING domain [76]. The authors employed heterologous expression systems approach similar as for PbBSMT to verify (a) the signal sequence in yeast and (b) in *E. coli* the E3-ubiquitin ligase activity. However, while for the in vitro activity an enzymatic function could be assigned, the in planta target remains unknown.

Since effectors play a role in defense suppression, Chen et al. [77] demonstrated that the *P. brassicae* effector PBZF1 interacts experimentally with a kinase from *A. thaliana* that was reported to affect the response to the clubroot pathogen. Overexpression of *SnRK1.1* in *A. thaliana* resulted in more resistant plants. Consequently, *PBZF* overexpression misregulated known target genes of SnRK1.1 and also resulted in higher susceptibility to the clubroot pathogen itself [77]. Remarkably, it was discovered that key KIN10 (=SnRK1.1) pathway genetic markers [78] are significantly similarly up- and downregulated in clubroot tolerant *AtOXR2*-overexpressing plants [51]. A significant number of these genes were likewise misregulated in plants overexpressing the *PBZF1* gene. The AtOXR2 protein was hypothesized to target the SnKR1.1. pathway to mediate the clubroot tolerance [51], although this needs to be confirmed experimentally.

Evidence for the involvement of proto-oncogenes in clubroot disease came from Bi et al. [63]. The comparison of genomes led to the identification of the so-called GPCR pathway, which stands for G-coupled protein receptor [64]. Treatment with an inhibitor of the GPCR pathway resulted in a reduction of disease symptoms.

Other *P. brassicae* proteins, such as the PbGH3 protein [27] and a putative homolog of indole-3-acetic acid dehydrogenase [79], have been implicated in the regulation of host hormone metabolism. For the former, an in vitro activity has been shown with the growth hormone IAA, whereas for the latter only a sequence prediction is available. The authors discuss that the potential IAA dehydrogenase could be a virulence factor since IAA is required for gall formation. These gene sequences came from the interaction of *B. napus* with a Canadian isolate of *P. brassicae* that can overcome the resistance of local cultivars. In this study, the NUDIX homolog identified by Daval et al. [43], as well as the PbBSMT sequence were also found [79]. Other hormone-related sequences that could be involved in the biosynthesis of CKs were identified in the genomes of *P. brassicae* (isopentenyltransferase genes) [27,62,80], but functional analyses were not performed on the encoded *P. brassicae* proteins. The NUDIX effector candidate was discovered during research into the effect of the microbiome on the transcriptome of *P. brassicae* and a host plant, *B. napus*, although functional analysis for this protein is lacking [43]. The gene for this protein was highly expressed under conditions where the condition of the microbiome led to high clubroot formation. A NUDIX protein of this type was already reported in a proteome dataset [3]. NUDIX effectors have been identified in other plant pathogens as well [81] and were therefore intriguing candidates in the mediation of the *P. brassicae*–host plant interaction.

Some effector candidates were identified via transcriptome studies rather than genomes, but the information from these datasets will be given in this context for completeness (Table 2). In the interaction between *P. brassicae* with *A. thaliana* a kinase called SSPb22 was discovered with a confirmed function and localization pattern [82]. In this report, several other candidates with putative effector motifs were reported, among them RxLR and Pexel motifs, and again, *PbBSMT* was identified [82].

A cysteine protease inhibitor could be another promising effector candidate [83]. Its activity has been confirmed indirectly since the resulting protein SSPbP53 was able to interact and inhibit cruciferous papain-like cysteine proteases [83]. Further, the *A. thaliana* mutant in *CYSTEINE PEPTIDASE 1* was more resistant to clubroot than the wild type [83] indicating an important role for this effector in the development of the disease.

Putative effectors with chitin-binding domains were also discovered [84], of which the secretion peptide was confirmed in yeast assays. Co-precipitation in vitro showed binding of these domains to chitin and to *P. brassicae* resting spores. Furthermore, the participation of these two proteins, PbCHi2 and PbChi4, in chitin-dependent activation of the immune response in *B. napus* was shown [84].

In tobacco, a collection of putative effectors has been analyzed for their localization and possible function [85]. Several were able to elicit PAMP-triggered immunity in this system, and some were found in the endomembrane system, which indicated their possible translocation into the host plant. Similarly, Chen et al. [86] identified several effector proteins that could induce cell death in tobacco leaves.

**Table 2:** Summary of *Plasmodiophora brassicae* proteins, including putative effectors, identified so far and their possible function.

Effector	Putative Annotated Function	Pathotype	Experimental Verification	Reference
Pro 1 (?) ^1^	Protease	SSI ^2^ Williams ^3^ P3	In vitro protease activity shown Treatment of plants resulted in better colonization	[70]
PbBSMT	SABATH-type methyltransferase	SSI e3 NZ field isolate SSI Williams P3	In vitro conversion of SA, BA, and AA to their methylester Transgenic *A. thaliana* plants are more susceptible to pathogens including *P. brassicae* and have higher Me-SA vs. SA content	[71,73,74]
PbCyp3 (?)	Immunophilin	SSI e3	Heterologous expression in *Magnaporthe oryzae* mutant restored virulence on rice	[72]
PbRING1	E3-ubiquitin ligase	SSI e3	E3-ubiquitin ligase activity confirmed in vitro Heterologous expression in yeast confirmed signal peptide function	[76]
PBZF1	RxLR effector	Chinese field isolate Present in SSI e3 and other isolates from databases	Physical interaction with kinase SnRK1.1 Heterologous expression in *A. thaliana* caused plants to be more susceptible to *P. brassicae*	[77]
SSPbP22	Kinase	SSI Williams P3	Kinase activity determined in vitro and protein modeling	[81]
SSPbP53	Cysteine protease inhibitor	SSI Williams P3	Interaction with and inhibition of cruciferous papain-like cysteine protease *A. thaliana* mutant in *CYSTEINE PEPTIDASE 1* more resistant to clubroot	[82]
PbChiB2 PbChiB4	Chitin-binding domain carbohydrate-binding module family 18	SSI Williams P3	Co-precipitation showed in vitro binding to spores of *P. brassicae* and chitin	[83]
GPCR pathway	G-protein coupled receptor pathway	SSI ZJ-1	Treatment with GPCR inhibitor resulted in reduced symptoms	[64]
NUDIX_hydrolase	NUDIX effector	eH, Somé et al. based P1	No experimental confirmation	[43]
PbGH3	IAA conjugating enzyme	SSI e3	In vitro conjugation of IAA to various amino acids	[27]
Indole-3-acetic acid dehydrogenase	Indole-3-acetic acid dehydrogenase	CCD based P5X	No experimental confirmation; Predicted function in IAA synthesis	[79]
Chitin synthase	Chitin synthase	SSI e3	Sequence prediction	[27]
PbTPS ^4^	Trehalose-P-synthase	SSI e3	Indirect by identification of trehalose in resting spores	[87]

^1^ (?) no classic effector in the sense of altering plant defense; rather factor needed for colonization; ^2^ abbreviations: AA: anthranilic acid; BA: benzoic acid; CCD: Canadian Clubroot Differential; IAA: indole-3-acetic acid; Me-SA: methylester of SA; NZ: New Zealand; SA: salicylic acid; SSI: single spore isolate; ^3^ classification system used; ^4^ not from an -omics study, but was the first gene fragment with an annotated function.

Some of the effector candidates identified have only been functionally evaluated in transient expression systems. Others have been predicted solely on bioinformatic sequence motifs. The latter group will presumably also contain “false positives”. Further identification of effector candidates therefore needs a different strategy not only based on effector motifs known from other plant pathogens. To summarize the numerous questions remaining: Where are the effectors localized in planta? Some data stem from heterologous transient expression in tobacco [38,85,86], which is not a transformation system close to a host of *P. brassicae*. Others have employed the host plant *A. thaliana* [73,74] to address the question of whether they are functional not only in vitro but also in planta. Since *P. brassicae* can so far not been routinely transformed, this is a central question that will remain. Some in vitro experiments may not be able to tell the exact substrate in planta which must be determined in order to properly comprehend the biological role of an effector. Which more closely related systems for heterologous expression can be used instead of tobacco? Some ideas have been discussed on possible cultured surrogate systems to study the effectome of *P. brassicae* in the future [88]. Among these are probably more closely related systems using fungal pathogens such as *Verticillium dahliae* or transient leaf transformation, but not using tobacco, instead using the host *A. thaliana* together with a hemibiotrophic bacterial pathogen.

### 4.2. Genomes from Host Plants Can Be Used for Functional Analyses

While genomic information for *P. brassicae* is gained from a small genome, this is not the case for the host plants. The genomic information is especially of interest for crop plants and not so much for the model *A. thaliana*, since such information can be used to obtain candidates to breed clubroot-resistant cultivars [89]. The authors review the feasibility of different types of genomic but also transcriptomic information for obtaining information that can be useful to breeders. They cover “…the impact of traditional marker-assisted selection-based breeding for clubroot resistance, and then discuss how -omics approaches have contributed to the (1) detection and genotyping of genome-wide SNP markers linked with clubroot resistance genes or quantitative trait loci, (2) understanding of host resistance mechanisms upon *P. brassicae* infection, and (3) acceleration of resistance breeding by identifying and characterizing candidate genes, especially those with differential efficacy against new pathotypes of *P. brassicae*”. Methods that have been used encompass genotyping by sequencing, high-density single nucleotide polymorphism arrays, genome-wide association studies, but also transcriptomic approaches such as (bulk-segregant) RNAseq analysis and microarrays when available for the host plant type.

### 4.3. Methylome and Epigenetic Regulations

Transcriptional regulation is among the well-accepted possibilities for differential gene expression (see Section 5). However, it is more and more recognized that epigenetic changes also play a role in the regulation of complete genomes. Consequently, the methylation pattern of DNA is involved in many regulatory processes. Specific sequencing techniques, such as bisulfite sequencing [90], can be used to identify the methylome.

A first link between an epigenetic factor and clubroot disease was published by Ludwig-Müller et al. [91], showing that a mutant in LIKE HETEROCHROMATIN PROTEIN 1 (LHP1), at that time isolated as a mutant defective in indole glucosinolate levels, was more tolerant to the disease.

Epigenetic variation in *A. thaliana* conferred a quantitative disease resistance to clubroot via transgenerational effects [40]. The involvement of DNA methylation in the development of the clubroot disease was supported by the reduction of the disease index in the *A. thaliana* mutant *ddm1* which has an altered methylation pattern. Other variations between ecotypes could perhaps be explained by different methylation patterns, for example in regulatory promoter elements.

Long non-coding RNAs (lncRAs) can also cause epigenetic regulations [92]. Such lncRNAs have been investigated in the response of *B. napus* to *P. brassicae*. Overall, 24 differentially expressed lncRNAs were identified in the interaction of resistant and susceptible *B. napus* lines and *P. brassicae* [93]. The link between these and chromosome A08, which is known to harbor a quantitative trait locus conferring resistance to different *P. brassicae* pathotypes, could be used for future studies including resistance responses. However, certain lncRNAs have been discovered to be miRNA targets and thereby constituting posttranscriptional regulations (see Section 5.1) [93]. Zhu et al. [94] integrated sequences of lncRNAs and mRNAs during their investigation of *B. campestris* and *P. brassicae*. They provided evidence for a regulatory network between mRNAs and lncRNAs.

## 5. Transcriptome and Posttranscriptional Regulations

**Figure 4:**
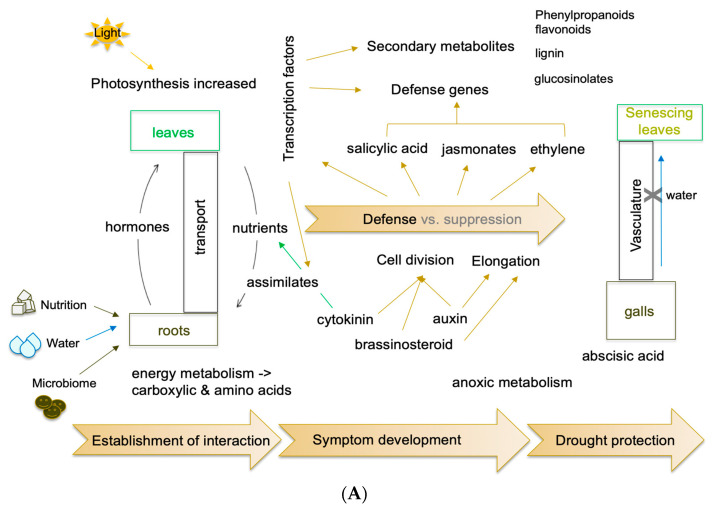

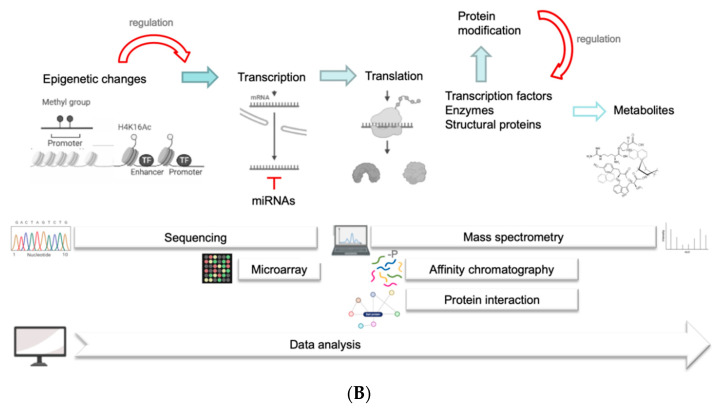
(**A**)**.** Model for molecular alterations in clubroots between the leaves and roots of host plants. These are generalized for different hosts (*A. thaliana*, various *Brassica* species) and compiled for different approaches to -omics data. (**B**)**.** The regulatory processes, methods, and techniques are shown in the lower part of the figure. Some cartoons were taken from the free version of Biorender (last accessed 23 April 2022).

The transcriptome comprises, theoretically, all transcripts that are expressed at a defined time point. However, low-abundant transcripts will be ignored in many experiments, and they must be addressed in more targeted approaches. Furthermore, the choice of meaningful experimental parameters is very important. These can be differential treatments and/or different time points during growth and development. This appears to be rather simple to determine in studies with a single organism, but when two or more organisms interact, the search for such experimental conditions such as the specific time points becomes critical. For example, the early interaction is important for determining the type of interaction, susceptible or resistant/partially resistant, but transcripts connected to later infection pathways are not expressed or at low rates [30]. Genome analyses were also complemented with transcriptomes from various developmental stages of the disease and also stages of the pathogen itself, such as isolated plasmodia or resting spores [27,49,62,64]. The results of the different metabolic and regulatory processes are summarized in Figure 4.

Over the years, approaches such as ESTs, (subtractive) cDNA libraries [23,24,25,26], microarray [2,30,31] and RNAseq techniques (e.g., [4,5,20,33,34,35]) have been used. Microarrays are the only method limited to detecting differences between plant tissues and were among the experiments that yielded the first information on global gene expression changes (e.g., [2,30,31]). All other techniques detected mostly the more abundant *P. brassicae* transcripts. With the reduction of costs, RNAseq is now the method of choice, but it requires competent bioinformatics for evaluation.

Comparing different biological functions with large datasets could help to understand interactions of pathways that are not obvious. For example, the transcriptomes of AtOXR2 and AtKin10 overexpressing plants have been compared to the transcripts of *A. thaliana* plants overexpressing the *P. brassicae* effector PbZF1, revealing a possible link between the role of AtOXR2 in tolerance induction and the AtKIN10 pathway targeted by the *P. brassicae* effector [51]. To back this claim, a statistically significant overlap was identified. The plethora of datasets is used through platforms such as Genevestigator or EFP browser [95,96] which give access to plant experiments. For the *P. brassicae*–plant interaction such experiments are not included in these databases as yet. Therefore, it is more complicated to compare different datasets.

Gene families were investigated in such large data approaches such as UDP-glucosyltransferases [97], sugar transporter families, including STP and SWEET [98,99], invertase [100], chitinase [101], auxin conjugating *GH3* genes [102], pectin-related genes [103], as well as functional pathways, e.g., flavonoids [38,104] or glucosinolates [105]. Other defense-related pathways including hormones will be discussed below (see Section 5.4).

Another challenge is that many different developmental stages co-exist in clubroots with even non-infected cells present. While data evaluating an entire organ can indicate trends when genes are strongly expressed, others might be diluted simply because the host tissue contains infected cells next to uninfected cells and also different developmental stages of *P. brassicae* at one developmental time point (examples can be found e.g., in [21]). Such developmental stages include small plasmodia in small host cells, dividing plasmodia, and growing plasmodia in already large host cells. Finally, one can find sporulating plasmodia that are about to form the resting spores, which are the stage where, at least in the host cell, possibly not much metabolism is taking place. Therefore, Schuller et al. [32] performed a microarray analysis on cells containing distinct stages of *P. brassicae* with laser dissection microscopy. As an outcome, several pathways that have been previously overlooked in such studies were identified, among them a role for brassinosteroids in clubroot development. Ludwig-Müller [106] evaluated bioinformatically co-expressed genes after this investigation and identified some promising candidates for further research.

### 5.1. miRNAs

microRNAs are small non-coding RNAs encoded in the genome and enzymatically cleaved to yield regulatory miRNAs of various sizes. These regulate their target mRNAs by hybridization and targeted degradation [107]. The expression pattern of miRNAs in response to *P. brassicae* was studied in oilseed rape (or canola) at two time points after inoculation, one early and one later time point, with different developmental stages of *P. brassicae* present [36]. For the differential regulation of miRNAs, it needs to be anticipated that the corresponding mRNA will be adversely regulated since high expression of a miRNA will result in the degradation of the target and vice versa. Differentially expressed miRNAs were confirmed by qPCR and their RNA targets through degradome analysis and through the analysis of the potential cleavage sites for the miRNAs by RLM-RACE. Targets for the miRNAs include transcription factors (TFs), hormone-related genes (for example auxin response factors, ARFs), as well as genes associated with plant growth or stress response regulation such as cytokinin and auxin/ethylene response elements. Novel miRNAs were identified in the differentially regulated set of another important crop plant, Chinese cabbage (*B. rapa*) using similar experimental approaches [108]. Extending these findings to resistant and susceptible cultivars of *B. napus* to *P. brassicae* identified several specific miRNA–target RNA pairs for the resistant reaction [109]. Consequently, specific miRNAs were identified that regulate disease tolerance genes of the TIR-NBS family in *B. napus* [110].

### 5.2. Nutrition

The formation of galls is also influenced by the plant’s nutritional status. Differential regulation of various metabolic pathways can be discovered in many transcriptome datasets. These include all aspects of energy metabolism since the formation of a strong metabolic sink is the essential feature that occurs throughout gall development (Figure 4). Photosynthesis products from the leaves accumulate here together with other nutrients. Among these are amino acids which are also precursors for secondary metabolites. From such datasets, functional categories of proteins involved in nutrient transport or availability have been extracted [98,99,100].

The developing gall is a site of extensive carbohydrate consumption and the redirection of sugars is dependent on many genes including modifiers of cell development, as reviewed by Malinowski et al. [111]. Invertases that catalyze the cleavage of saccharose were upregulated in the clubroot interaction. When tissue-specific expression of an invertase inhibitor protein was utilized, this resulted in a reduction of gall size [100]. Since the sugars need to be translocated into the plasmodia the upregulation of two different classes of sugar transporters was reported [98,99]. Due to the presence of gene families, and that mutants would affect the plant’s overall sugar metabolism, transgenic or mutant approaches are more difficult here. Siemens et al. [100] therefore proposed tissue-specific reductions as a solution. Genes encoding for sucrose degrading enzymes were also detected in the comparison of a susceptible and partial resistant interaction [31].

Another interesting aspect is the energy status of such clubroots. The switch from aerobic to anaerobic metabolism was already noted in [31] and thought to occur due to the accumulation of excessive carbohydrates at the infection site. These modifications occurred in both a partial resistant and a susceptible interaction [31]. There was also an enrichment of genes implicated in hypoxia metabolism in transcriptomes of enriched single cell populations [32]. This observation was corroborated in later work by Gravot et al. [112] demonstrating that the hypoxia response in *A. thaliana* supported the development of clubroots. Specifically, pyruvate decarboxylase gene mutants were altered in the response to the protist. They discovered an elevation of a group of hypoxia-regulated core genes in infected vs. control roots using the dataset from [80]. Another connection between hypoxia-regulated genes and plants with an altered response to clubroot was reported in [51], where the authors found overlaps between hypoxia-regulated transcriptional datasets and *A. thaliana* mitochondrial gene *OXR2* overexpressing plants.

The effect of nutrient (nitrogen) supply on the transcriptome of resistant and susceptible *B. napus* was analyzed [113]. The salicylic acid (SA) response was induced irrespective of the nutrient treatment and genotype, whereas the low nitrogen-driven resistance was dependent on low expression of nitrate uptake and nitrate reductase. Genes involved in auxin transport and signaling showed a similar response [113].

### 5.3. Cell biology and Growth Promoting Hormones

To establish the above-mentioned sink in clubroots (Section 5.2), nutrients need to be transported from the shoots to the infected roots, and a signal is required in the clubroots for this. Since cytokinins (CK) regulate such processes in healthy plants during development [114], they are also thought to play a role in disease development. In addition to their function in sink control, they are also important players in the establishment of the growing tissue by cell division [114]. Plant hormones regulate many features that are important for the growth of the clubroots in *A. thaliana* and other *Brassica* species. The cell cycle is important, and once the pathogen has established itself in the root cortex, cell enlargement occurs. Plant hormones orchestrate both responses. Cell cycle control is mediated mainly by CKs, brassinosteroids (BRs), and auxin [115]. The latter is also a major regulator of cell enlargement [115]. Manipulation of these hormones can change gall size, albeit it is usually critical to assess due to its effects on the entire plant growth [2,32,101,105].

Cell cycle genes were identified as critical mediators for the reprogramming of growth of root galls in datasets described for *A. thaliana* [20]. Further, Malinowski et al. [80] investigated cytokinin biosynthesis-related genes in developing galls based on the transcriptome from [20]. Their findings did not completely support a role for cytokinins in pathogen–plant interactions and growth conditions as strongly as those found in *A. thaliana* plants overexpressing cytokinin oxidase/dehydrogenase genes [2]. The outcome of experiments might not always be comparable since they strongly depend on the growth/light conditions, the pathotype, and the host genotype.

In a growing plant, auxin and brassinosteroids are responsible for cell division, but also for cell elongation [116]. Many genes encoding proteins in BR synthesis, metabolism, and perception were found to be differentially regulated in the transcriptome dataset from individual cell populations with different plasmodial stages. BRs are involved in club growth since plants mutated in the receptor *BRI1* as well as plants treated with a BR biosynthesis inhibitor showed reduced gall size [32].

Auxin homeostasis genes are differentially regulated in various datasets (e.g., [2,31,32,43]), and this has been exploited in some follow-up studies. Among these are the so-called *GH3* genes based on the dataset of [2], encoding for proteins involved in the conjugation of IAA to amino acids [117]. *GH3* double mutant plants are marginally more susceptible to clubroot, according to functional approaches [102]. Auxin perception and transport are also important since the treatment of infected plants with the respective inhibitors caused the growth of galls to be reduced [102]. Taken together, this research found that two types of auxin receptors, as well as potassium channels, play a role in the elongation response.

Auxin has also been linked to cell wall remodeling [116,118]. Potassium transport has been indicative of acid growth-dependent cell elongation, also involving expansins [102]. Cell wall elongation connected to expansins has already been postulated for the interaction of *A. thaliana* with *P. brassicae* on the transcriptome level [2] and was later confirmed with high upregulation of expansin genes in a susceptible interaction of *B. napus* with *P. brassicae* [119]. Interestingly, an expansin was also on the gene list that was altered after the overexpression of the *P. brassicae* effector PBZF1 in *A. thaliana* [77]. Other cell-wall modifying proteins are encoded by pectin methylesterases, which were analyzed for their role during clubroot development [103] based on the dataset from [80]. Functional analyses confirmed that the club development in a *pme18* mutant was altered, namely cell wall enlargement was reduced. Furthermore, the transcriptome data were supplemented by cell wall component analysis, which revealed a change in the content of methylated pectin [104].

Abscisic acid is a stress signal frequently linked to drought stress. In older root galls a strong increase in the number of ABA-associated transcripts was found [2,120]. These old roots are destroyed and therefore, the water transport capacities are diminished. Further work based on gene expression has not been done on ABA-related processes, but ABA was discovered in clubroots during later stages of infection in susceptible interactions (see Section 7).

### 5.4. Defense Responses and Defense Signals

Clues on defense pathways can be obtained from comparisons of 1. (partial) resistant vs. susceptible cultivars of the same plant species; 2. infected vs. non-infected roots on the same plant; 3. infection of different pathotypes of *P. brassicae* on one host root; 4. mutants vs. wild-type plants (Figure 2). Since in RNAseq studies also *P. brassicae* transcripts can be detected, they were also reported in some studies [33], although the majority of examples from the plant side will be discussed in this section.

The defense response of plants is often regulated by plant hormones [121]. For biotrophic pathogens, primarily SA is discussed, but evidence for jasmonates (JA) and ethylene has also been reported in clubroot interactions [111,119]. An early response to SA signaling and a later response to JA were found in the comparison of a partially resistant interaction with a compatible of *A. thaliana* [62,122]. In roots and shoots of *A. thaliana*, the infection resulted in the upregulation of the SA- and JA-regulated defense pathways, as well as the shikimate pathway [37].

In *B. napus* interactions with resistant and susceptible cultivars, the resistance response was mediated mainly by SA pathways [119]. Already early in infection, genes associated with pathogen-associated molecular patterns and effector recognition, SA signaling, pathogenesis-related genes, and cell wall modification were upregulated in resistant vs. susceptible roots [4]. The authors concluded that the recognition in the resistant line was already high during early infection, while recognition pathways were suppressed in the susceptible line [4]. A strong SA-dependent response in a root gall on a single root of *B. oleracea* var. *gongylodes* compared to a non-infected one from the same plant revealed strong induction primarily of SA-dependent defense responses and the authors concluded that a non-infected root reacted similarly to a resistant one under their field conditions [33]. They allocated these findings partially to the high expression of *PbBSMT* in the galls (4.1).

In the resistant *B. rapa* ssp. *rapeifera* (line ECD4 from the European Clubroot Differential), carrying resistance to clubroot, many typical resistance genes were annotated in the genome, and evidence for SA- and JA-related defense genes was revealed in the transcriptome [123]. Analyzing the genetic architecture by comparing the transcriptome of different resistant *Brassica* species with respect to their response to *P. brassicae* resulted in the identification of a major group of resistance genes, but also in transcripts encoding secondary metabolite pathways and lipid transport [124]. Obviously, the SA response is not responsible for the outcome of the disease in all interactions. A weighted genetic network was constructed that could lead to the identification of more target genes for subsequent analysis. The analysis of differentially expressed genes in *B. napus* during infection with virulent and avirulent *P. brassicae* pathotypes revealed no apparent pattern that would be induced by a more or less virulent pathotype [48].

Other authors identified, next to the activation of the SA-dependent defense response, ethylene as a signal in a comparative transcriptome analysis of rutabaga cultivars, another *B. napus* species, in response to *P. brassicae* [125]. The respective defense pathways were supported by the differential regulation of *WRKY* genes that are known as SA response transcription factors [126], and SA- as well as ethylene-responsive genes. In another *B. rapa* comparison of susceptible and resistant cultivars and *P. brassicae*, the general induction of defense pathways was confirmed but the authors found additional evidence for upregulation of ethylene and brassinosteroids in the resistance response, whereas the SA response was downregulated [127]. In addition, the role of SA and JA as defense signals was investigated in the comparison of susceptible and resistant *B. oleracea* var. *capitata* [5]. SA, JA, ethylene, and brassinosteroids were identified in Chinese cabbage (*B. rapa*) transcriptomes as defense signals [128]. In conclusion, there is a lot of evidence for SA signaling during many early defense responses, but also for the participation of JA at later time points, whereas a role for ethylene has only been suggested in some experiments. However, *A. thaliana* mutants in ethylene perception were more susceptible to clubroot [129]. Finally, brassinosteroids have been linked to the development of galls (see Section 5.3), as well as to defense responses.

Evidence for the involvement of secondary metabolite pathways in defense is most consistent for the phenylpropanoid pathway that leads to cell wall components, flavonoids, and other phenolics, among them SA. A strong regulation of the phenylpropanoid metabolism was found after inoculation of susceptible and resistant *B. napus* cultivars with the same *P. brassicae* isolate, where, in the latter, more time points showed an elevation of defense associated genes [38]. The pathways linked to lignin and flavonoids/anthocyanidin synthesis were similarly upregulated at early time points, i.e., in the root hair of the *A. thaliana*–*P. brassicae* interaction and were regarded as being of major importance [35]. A potential role for flavonoids based on the dataset of [2] was investigated, and PCR was used to confirm the transcriptome data [104]. In situ staining of root galls with a reagent that specifically stains flavonoids and sinapates showed differential accumulation patterns for both compound classes. In addition to the phenylpropanoid pathway, genes encoding for enzymes involved in terpene and glucosinolate pathways were upregulated in an early *A. thaliana*–*P. brassicae* interaction [35,105].

## 6. Proteomes of *Plasmodiophora brassicae* Inoculated Tissues

While transcriptome studies are easier to conduct, as seen by the amount of publications on the various methodologies, some proteome data on *A. thaliana* and *Brassica* species were published early on. However, like with transcriptome data, approaches for analyzing proteomes became more sensitive, resulting in a rise in the number of proteins discovered in such datasets. Furthermore, the quantity and quality of sequences in databases are rising, making peptide sequence annotation easier. Proteins reported in datasets such as differentially expressed confirm transcriptome data (see Section 5) and complement metabolomes [59] (see Section 7). Therefore, the results will be discussed only briefly here since the pairs used for comparisons were often similar [6]. In addition, proteomes were acquired from single conditions [3]. 

Pioneering work was done in *A. thaliana* [3] and *B. napus* [130] and *P. brassicae* infections where only a small number of proteins were identified, including cell cycle and cell wall modifications [3] and hormone metabolism, calcium signaling and ROS detoxification [130]. Proteome analyses at initial infection stages of Chinese cabbage confirm a role for SA and JA in defense [131]. Other studies utilizing *B. napus* revealed a resistance response connected with proteins involved in secondary metabolite pathways [132] or, for *B. oleracea*, an involvement of ABA in the resistant interaction based on the proteins discovered [6]. A proteomics experimental strategy to elucidating Rcr1-mediated resistance in *B. rapa* found association with proteolysis and a unique signaling mechanism that needs further investigation [133].

Two studies [134,135] describe the protein profiles of *B. rapa* in response to *P. brassicae* utilizing a more targeted high-throughput functional proteomics technique combining iTRAQ affinity chromatography with mass spectrometry for large molecules. This results in the extraction of phosphorylated proteins, which can provide information about the post-transcriptional changes in plant proteins caused by *P. brassicae* interaction. During the secondary phase of the infection cycle, proteins involved in energy and lipid metabolism, plant defense, cell wall modification, and hormone production and signaling were discovered to be differently expressed [135]. The role of brassinosteroid biosynthesis was verified in this study. The other experiment revealed evidence for glutathione in the resistant and cytokinin production in the susceptible *B. rapa* interaction.

## 7. Metabolome Analyses of *Plasmodiophora brassicae* Inoculated Tissues

Metabolites are the end products of enzymatic reactions, which are catalyzed by proteins, and these are encoded by the transcripts. It is therefore evident that the metabolites are the end results of biochemical reactions, whereas the proteins can also encompass regulatory ones such as transcription factors or kinases.

“A hormone and proteome approach…” was the title of the first publication in this methodological aspect of clubroot research, but Devos et al. [3] reported only on a few compounds and proteins. Further work on hormones is still restricted to a more targeted approach since untargeted metabolome analyses usually do not find plant hormones that are present in lower amounts. Furthermore, such metabolite patterns have often been supported by transcriptome datasets. The presence of metabolites involved in oxidative stress [112] was also confirmed in the microarray experiment by Schuller et al. [32] who found a significant number of differentially regulated genes involved in anoxic metabolism. Another example is lipid metabolism, where several -omics techniques were used [49] which are discussed in greater detail here.

Scanning electron microscopy of *P. brassicae* infected roots in early publications showed the presence of lipids as putative storage vesicles in secondary plasmodia [136]. However, the analysis of lipid compounds is more complicated due to their chemical nature. While transcriptome approaches had detected differentially expressed genes in lipid pathways, their presence in the plant and/or pathogen was only demonstrated in the “multi-omics” approach using genomic, transcriptome, proteome, and (targeted) metabolome data to characterize the patterns of lipid biosynthesis associated pathways in *P. brassicae* [49]. The authors confirmed the presence of lipid-containing structures in resting spores, germinating spores, and primary and secondary plasmodia by Nile red staining. Physiological evidence for alterations in lipid patterns in infected vs. control roots of *A. thaliana* was previously reported using a TLC analysis approach with staining for triacylglycerol derivatives [137] and on a histological basis using Nile red [138]. Bi et al. [49] performed proteome and lipid analysis on isolated lipid droplets of *P. brassicae*. They identified over 200 proteins associated with these lipid droplets and their transcriptome. Further, lipid analysis was done to identify the free fatty acid composition. These analyses revealed that *P. brassicae* lipid droplets are rich in arachidonic acids [49]. Fatty acid biosynthesis and metabolism were discovered by KEGG pathway analysis for putative functions. This is probably the most comprehensive analysis for one group of metabolites, deriving from the pathogen in clubroots. Schwelm et al. [27] speculated that part of the lipid biosynthesis occurs in *P. brassicae*, but that the pathway is not complete in the protist, and that precursors of lipids would be needed for a complete pathway. Such a feature was discussed as a hallmark of biotrophic pathogens that may not require a complete pathway if the host can supplement missing parts.

Secondary metabolites have been studied more in depth using targeted approaches, such as indole-derived defense compounds, and these reported the production of at least 45 different metabolites in *B. napus* infected by *P. brassicae* [139]. A comparative mapping of quantitative trait loci associated with resistance was carried out using metabolome profiling and pathogen quantification in a segregating progeny of *B. napus*. Distinct metabolic modules were discovered, and the glucosinolate gluconasturtiin was identified in addition to two as yet unidentified compounds within them [140]. This is an intriguing example of metabolic profiling (=metabolomics) and genetics working together. The results for an untargeted analysis on *B. rapa* ssp. *pekinensis* roots were not so clear with respect to the metabolites detected in the different interactions, but glucosinolates appeared to be typically higher in infected roots compared to controls [141]. It is clear that more analyses are needed to complement the transcriptomes.

A more global hormone analysis was done by Prerostova et al. [39] comparing control and infected roots and leaves of resistant and susceptible *Brassica napus* plants. Two groups of growth promoting hormones, auxin, and CKs, as well as defense compounds jasmonates (JA) and SA and the abiotic stress hormone ABA were determined. The patterns reported were quite complicated and accompanied by transcriptional data for hormone-related genes based on qPCR, not RNAseq, so they are not reported in the above transcriptome section. It is noteworthy that this work is one of the few also including leaf materials of infected and control plants. Altogether, the work showed a positive correlation of gall growth with auxin and CKs, as well as upregulation of defense hormones in the resistant cultivar at earlier time points of the study. In leaves and roots, but not galls, ABA was induced during the late response [39] which corresponds to the ABA-related genes that were reported in transcriptomes [2,120]. Furthermore, a comparison of a resistant with a susceptible line and subsequent analysis of their hormonal profiles in addition to the transcriptional response linked ABA to a function in the late susceptible response in roots of *B. rapa* [127]. In addition to growth-promoting hormones and abiotic stress signals, the defense compounds JA, SA, and the ethylene precursor ACC were determined. A suppression of JA and SA in the resistant line at late stages was found, but their upregulation in the susceptible line [127]. In another study comparing resistant and susceptible *B. rapa* ssp. *pekinensis* the SA-dependent resistance pathway was induced in the resistant interaction based on phytohormone analyses, while the susceptible genotypes were higher with growth promoting hormones auxin and CK [141], confirming previous reports.

## 8. Potential Role for the Microbiome

Microbiome studies have demonstrated that the former can have a significant impact on the latter in other plant microbe interactions [142]. Since these microbiome studies result in a plethora of organisms belonging to pro- and eukaryotes (e.g., [43]), these findings will not be described in detail. Rather, the various conditions that were used to examine the microbiomes associated with clubroot disease will be reviewed. Individual soil microbes can influence the outcome of the clubroot disease, of which most are isolated from soils or the rhizosphere, and such effects of disease reduction are interesting for biocontrol mechanisms (e.g., [143,144]). Such microbes can also be present in more complex soils, so it is of interest to understand this specific aspect better in more detail.

A study employing symptomatic and asymptomatic roots of *B. napus* from the same field gave evidence for a strong alteration in the microbiome between such roots [42]. The number of microbes, both bacteria and fungi, associated with the roots showing disease symptoms was lower than those associated with healthy roots. A more complex setup was investigated by Daval et al. [43]. They compared two different *B. napus* cultivars with different resistances to *P. brassicae* and three soils harboring high, medium, or low microbiota diversities and levels of richness. Clearly, the soil microbiota had an impact on the outcome of the interaction in the genotypes as well as over time. Other treatments and experimental conditions that resulted in microbiome modifications included different rotation patterns for fields to reduce the clubroot incidence [44], and various biocontrol trials such as rice straw, which stabilizes the microbiome and reduces clubroot [45], and the effect of fungicides [47]. For the latter condition, one would, of course, assume that their application will alter the microbes’ composition, but it is nevertheless necessary to analyze such predictions.

When such experiments are taken to the field alterations in the microbiome were seen when the plants were treated with *Streptomyces alfalfae* to reduce clubroot symptoms [46]. Such considerations are critical for long-term treatments in nature and their effect on the natural microbial populations. Under natural field conditions, the comparison between infected and asymptomatic roots of *B. napus* also showed differences in the endobiome of roots [42]. The microbial population and its relative abundance in the asymptomatic roots in this study was far higher than that in the symptomatic roots, and many microorganisms in asymptomatic roots have biological control and plant growth promotion functions that could alter the clubroot symptoms. It should be possible to identify even novel microbes originating from natural conditions that are suitable for biocontrol, using such methods.

As already mentioned, not only the root-associated microbes are altered, but also the endophytic community has been analyzed for changes in clubroot infected and control tissues of *B. juncea* [145]. There were distinct differences in the comparison, such as the dominance of *Pseudomonas* at the genus level in clubroots, whereas *Rhodanobacter* was the dominant genus in healthy roots, but there were also some parallels [145].

## 9. Status of Other Plasmodiophorid Genomes

**Figure 5:**
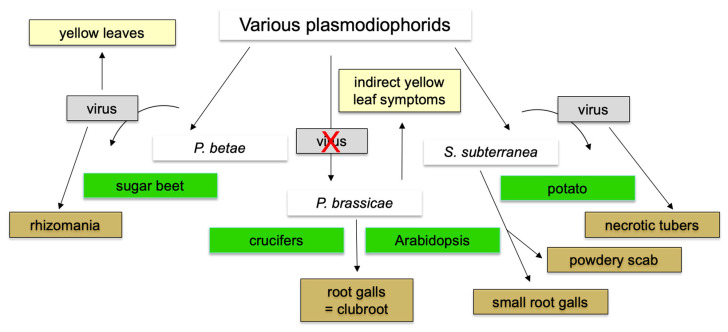
Comparing three plant pathogenic plasmodiophorids, *P. brassicae*, *P. betae*, and *S. subterranea* for their disease symptoms or virus transmitter capability. Only those with currently available genome information are shown. The red cross indicates no virus transmission.

The systematics for plasmodiophorids, including the pathogenic groups, has been recently again updated [146]. In addition to *P. brassicae*, two other plasmodiophorid genera are known that infect important crop plants (Figure 5). *Polymyxa betae* and *P. graminis* confer the disease symptoms to their hosts by transmitting viral diseases as vectors [147]. *Spongospora subterraneana* is also a transmitter for a viral disease, but also causes root galls on their host, albeit much smaller than *P. brassicae* [147,148]. Interestingly, for *P. brassicae* there is no evidence that it is a vector for the transmission of viral diseases since only the clubroot symptoms have been described. Other pathogenic genera e.g., *Maullinia braseltonii* invade host plants with low economic importance, such as brown algae [149,150].

In a first approach with another plasmodiophorid organism, Mutasa-Göttgens et al. [151] generated 11 *Polymyxa betae* Expressed Sequence Tags (EST) using a phagemid library of cDNA clones from infected sugar beet roots. The same strategy was applied for *Polymyxa graminis*, generating four EST [152] and for *P. betae*, generating 76 *P. betae* EST as well as 120 sugar beet ESTs upregulated during the different stages of the infection process [153]. Following that, cDNA from five rhizarian species including *P. brassicae* and *Spongospora subterranea* ESTs was sequenced using the 454-pyrosequencing technology [154]. Bulman et al. [24] employed dual culture systems to add some more sequences from *S. subterranea* and *P. brassicae* that were differentially expressed. With the genomes of *S. subterranea* in 2018 [155] and *P. betae* in 2019 [156] available, the molecular biology of plasmodiophorids can now move further. Key genes identified in the *P. brassicae* interaction could also be important for other plasmodiophorid–host interactions. While the *S. subterranea* genome is still in a draft status [155], the genome of *P. betae* has been further improved and the mitochondrial sequence was also annotated and published [156]. The latter revealed that there are differences between *P. brassicae* and *P. betae* in genome size and thus also in the number of proteins that are encoded [27,156].

More protist genomes are needed for comparative analyses that fully encompass their diversity. Indeed, there are other pathogenic plasmodiophorids than the ones mentioned above, among them organisms able to colonize algae or oomycota, and therefore being adapted to a different lifestyle, for example in water [157]. Genome data can provide insight into such adaptations.

## 10. Conclusions

The period of -omics research in clubroot started in the early 2000s. There is a large amount of data that were gathered over the years that followed, and the new data are increasing rapidly. Further, the genomes for *P. brassicae* pathotypes have added to the complexity. Previously, subtractive cDNA libraries could only discover a few genes from the pathogen, but since this was not exhaustive, one must recognize that the large number of *P. brassicae* genes identified was attributable to the genomes. It would be ideal if such datasets are readily available to the community. The plant side is better covered, but also here the availability of the datasets does not necessarily guarantee their widespread use, such as for comparisons.

While effectors have been predicted using bioinformatics on genomes, more methods for their functional analysis are required. Other gene predictions need to be carried out as well. Since the sequence of *P. brassicae* differs significantly from other organisms, the functional prediction should be complemented by other methods. These can include the modeling of protein structures based on web-based tools such as SWISS MODEL or others (e.g., [158]). Several of the above-mentioned effectors, for example, have been modeled and the functional prediction could be supported by other experimental approaches [71,76].

Heterologous transformation will remain a method of choice since the clubroot pathogen up to now cannot be routinely transformed. Expression studies in microorganisms, such as *E. coli*, have led to the identification of a putative protease function during early infection [70], a SABATH-type methyltransferase (PbBSMT) that can methylate the defense compounds salicylic acid (SA), benzoic acid and anthranilic acid [71] and a GH3-type auxin conjugate synthetase [27]. The expression of a gene encoding a RING domain protein has also given some clues to its function [76]. The function of the secretory peptide of the PbBSMT protein was validated in vitro using a heterologous artificial yeast system [73]. In addition, the heterologous expression of *P. brassicae* candidate genes in respective mutants of fungi has been done and confirmed the role of another *P. brassicae* protein in vitro [72] (Table 2).

Transient transformation of tobacco leaves is another potential strategy for heterologous transformation; however, this is possibly an even more artificial system, although results can be generated relatively quickly, similar to microbes. The gene encoding PbBSMT was transiently expressed in tobacco and the authors showed a higher susceptibility to a leaf pathogen, *Pseudomonas* [62,73]. Finally, the genes can be heterologously transferred into host plants like *A. thaliana*. This was done to show that overexpression of *PbBSMT* caused more susceptible plants [74] as well as higher levels of the SA methylester (Me-SA) vs. SA [73]. Other examples used a larger-scale experimental approach with tobacco leaves and transient transformation of numerous effectors [84,85]. These also led to some information on their localization but were all rather artificial involving a no-host organism and also the “wrong” organs, namely leaves. As discussed in [88], other model systems for functional analyses are therefore required.

## Figures and Tables

**Table 1:** The approaches historically and currently used to analyze transcripts on a more global level. The references are selected for a given technique and combination, but they are not exhaustive.

Method	Achievement	Reference
(Random) PCR cloning	Genomic DNA (fragments)	[28] Bulman 2007
Subtractive cDNA library	76 gene sequences from *P. brassicae*	[23,28]
Suppressive subtractive cDNA library	Ca 60 *P. brassicae* sequences	[25,26]
Dot blot and qPCR	Larger-scale expression profile of >100 genes during primary and secondary zoospore development	[29]
Microarray ^1^	Role of cytokinins Early infection Partial resistance	[2,30,31]
Laser capture microdissection coupled to microarray	Role of brassinosteroids	[32]
RNAseq ^1^	Host plant genes *P. brassicae* genes	[5,20,33,34,35]
miRNA expression	Host plant	[36]

^1^ For all different comparisons described in this review see Figure 3.

## Data Availability

Not applicable.
